# *Citrus limon* (L.) Osbeck Fruit Peel Extract Attenuates Carbon Tetrachloride-Induced Hepatocarcinogenesis in Sprague-Dawley Rats

**DOI:** 10.1155/2024/6673550

**Published:** 2024-01-02

**Authors:** Alex Boye, Ernest Amponsah Asiamah, Orleans Martey, Frederick Ayertey

**Affiliations:** ^1^Department of Medical Laboratory Science, School of Allied Health Sciences, College of Health and Allied Sciences, University of Cape Coast, Cape Coast, Ghana; ^2^Department of Biomedical Science, School of Allied Health Sciences, College of Health and Allied Sciences, University of Cape Coast, Cape Coast, Ghana; ^3^Department of Pharmacology, Center for Plant Medicine Research, Mampong-Akuapem, Eastern Region, Ghana; ^4^Department of Phytochemistry, Center for Plant Medicine Research, Mampong-Akuapem, Eastern Region, Ghana

## Abstract

**Background:**

Traditional herbal medicine practitioners in the Ashanti region of Ghana use the fruit peels of *Citrus limon* (L.) Osbeck (*C. limon*) in preventive and curative treatment of many cancers including liver cancer. This ethnobotanical claim remains to be verified scientifically. *Aim of the Study*. This study investigated prophylactic hepatoprotective and anti-HCC effects of *C. limon* peel extract (LPE) in CCl_4_/olive oil-induced HCC-like rats.

**Materials and Methods:**

After preparation of LPE, it was subjected to phytochemical screening using standard phytochemical methods. A total of 30 healthy adult male Sprague-Dawley rats (weighing 150-200 g) were randomly assigned into six groups of 5 rats each. Rats in the control group received olive oil (5 mL/kg *ip*) twice weekly for 16 weeks. Rats in the model group received CCl_4_/olive oil (2 mL/kg, *ip*) twice weekly for 16 weeks. Rats in capecitabine (10 mg/kg *po*) and LPE (50, 100, and 200 mg/kg *po*) groups received CCl_4_/olive oil (2 mL/kg, *i.p*) in the morning and their respective treatments in the afternoon twice a week for 16 weeks. Rats in all groups had free access to food and water *ad libitum*. Body weight and survival rates were monitored. Rats were sacrificed under deep anesthesia, blood was collected, and liver and other organs were isolated. Aspartate transaminase (AST), alanine transaminase (ALT), alkaline phosphatase (ALP), gamma-glutamyltransferase (GGT), prothrombin time, bilirubin, C-reactive protein (CRP), alpha- (*α*-) fetoprotein (AFP), and liver histology were assessed.

**Results:**

Alkaloids, tannins, flavonoids, terpenoids, and saponins were detected in LPE. Model rats demonstrated increased serum levels of AFP, CRP, ALP, GGT, ALT, and AST, prothrombin time, total bilirubin, direct bilirubin, blood lymphocyte, and monocyte counts, but decreased serum albumin and total protein compared to control rats. Unlike the control, model rats demonstrated fat accumulation in periportal and centrilobular hepatocytes and neoplastic transformation. Semiquantitation of periodic acid Schiff- (PAS-) stained liver sections showed decreased glycogen storage in hepatocytes of model rats compared to control rats. Compared to the model, LPE treatment protected against CCl4-induced hepatocarcinogenesis, which was evidenced by decreased AFP, CRP, liver enzymes, total and direct bilirubin, prothrombin time, and blood lymphocyte and monocyte counts; attenuation of fat accumulation; and increased glycogen storage, albumin, and total protein.

**Conclusion:**

LPE abates CCl_4_-induced hepatocarcinogenesis by attenuating liver inflammation and improving metabolic, biosynthetic, and detoxification functions of the liver. The prophylactic hepatoprotective and anti-hepatocarcinogenic effects of LPE are attributable to its phytochemical composition raising hopes of finding potential anticancer bioactive compounds from *C. limon* fruit peels.

## 1. Introduction

Hepatocellular carcinoma (HCC) accounts for 85-90% of primary liver cancer. Globally, HCC is the fourth most common cause of cancer-related deaths. The majority of HCC cases are diagnosed in the advanced stage in view of its incipient nature; therefore, late diagnosis narrows both treatment options and median survival (approximately 6 to 20 months) postdiagnosis [1]. Among the available therapies (transarterial chemoembolization, radiotherapy, surgical resectioning, chemotherapy, small molecule-based therapies, liver transplantation, etc.), only surgical resection and orthotopic liver transplantation are curative [2]. These curative anti-HCC therapies are frothed with many problems. For example, orthotopic liver transplantation is challenged as the inflow of donor livers is outpaced by the growing number of patients who are added to the elective liver transplant waiting list [3]. Additionally, survival rate and quality of life postcurative treatment are poor alongside high recurrence rates (80% at 5 years) [4]. Also, systemic adverse reactions are associated with chemotherapy. Adverse outcomes associated with currently available anti-HCC therapies underscore the need for more efficacious and tolerable treatment options. In view of the inflammation-related pathogenesis of HCC [5], it is plausible to expect with certainty that natural products enriched with secondary plant metabolites that demonstrate both anti-inflammatory and antioxidant properties could be an invaluable source of effective therapeutics in the treatment of inflammation-mediated cancers, including HCC. Interestingly, ethnobotanical information shows that medicinal plants in the genus *Citrus*, family *Rutaceae* have wide applications in ethnomedicine in view of their rich and diverse secondary plant metabolites [6]. Indeed, these plants have been recognized in traditional medicine of many cultures, including that of Africa and Asia. Taxonomically, the genus *Citrus* is a taxonomic subunit of the family *Rutaceae*. The genus *Citrus* comprises seventeen species, including but not limited to *Citrus reticulata* (mandarin orange and tangerine), *Citrus sinensis* L. (sweet orange), *Citrus aurantium* L. (bitter orange), *C. limon* L. (Latin synonyms: *Citrus lemon* L. and *Citrus limon* L.), *Citrus paradise* M. (grapefruit), and *Citrus medica* [7]. Morphologically, the genus *Citrus* comprises plant species (shrubs and trees) with heights ranging between 3 to 15 m [7]. They possess leathery lanceolate leaves. The stems may have many branches with spines depending on the species. One of the most commonly used parts of *Citrus* plants is their fruits. They have flowers that develop in leaf axils. Each individual flower is pentapetalous with either white or red color. Their fruits are hesperidium berries. *Citrus* fruits find many applications in view of their nutritional and extranutritional (medicinal, pharmaceutical, and cosmetic) properties. Ecologically, *Citrus* species are native to warm and tropical ecological zones such as in Africa and the Mediterranean [7].

Different parts of *Citrus* plants such as leaves, fruits, seeds, and fruit peels are used in folklore medicine [8]. For example, fruit juice from *Citrus* spp. has demonstrated anti-inflammatory and antioxidant properties [9]. Likewise, a systematic review assessing medicinal properties of *Citrus* fruit juice highlighted the anticancer properties of *Citrus* fruit juice that have been demonstrated *in vitro* and *in vivo* [10].*C. limon* is the third most common *Citrus* fruit used in regions where it is grown. The fruit peel of *C. limon* constitutes almost half of the fruit mass. It is reported that juice from *C. limon* fruit exhibits anticancer properties [11]. For example, *C. limon*-derived nanovesicles were shown to have antiproliferative properties against a number of humanized cancer cell lines, including A549, LAMA84, and SW48 *in vitro*, and also suppressed CML tumors *in vivo* [11]. *C. limon* fruit juice exhibited cytotoxicity against promyelocytic leukemia cell line HL60 [12]. Likewise, juice from fruits of *C. limon* and other *Citrus* spp. exerted antiproliferative effects against several cancer cell lines *in vitro* [13]. Most research on *C. limon* in terms of their nutritional, extranutritional, and medicinal uses has overly focused on its fruit juice, seed, leaves, and flowers and has neglected the fruit peel. Interestingly, the peels of *C. limon* fruits harbor diverse secondary plant metabolites with anti-inflammatory and anticancer properties. *C. limon* fruit peels are rich in flavonoids and other phenolic compounds. In most communities in Asia and Africa, the fleshy part of *C. limon* fruit is eaten either raw or juiced while the fruit peels are discarded. In other traditional settings, the fruit peel is used as a livestock feed either freshly or dried. Also, the fruit peel may be used by local women to clean rusty and blackened cooking wares while other women in some parts of Africa use the fresh fruit peel in crude form to maintain vaginal hygiene [8].

An ethnopharmacological survey of medicinal plants used by traditional herbal medicine practitioners for cancer prevention and treatment in the Ashanti region of Ghana identified *C. limon* and *Citrus sinensis* as anticancer medicinal plants [8]. The report also indicated the use of fruits from the two *Citrus* spp. in the treatment of liver, breast, and prostate cancers. Although the ethnopharmacological survey identified the medicinal properties of these two *Citrus* species, the ethnobotanical anticancer claims of *C. limon* are yet to be verified.

Capecitabine, a 5-fluorouracil (5-FU) prodrug normally used to treat many solid malignancies inducing liver cancer [14, 15], was used in this study as a reference anticancer drug. Metabolically, capecitabine is biotransformed by hepatic enzymes into a number of metabolic intermediates with 5-FU being the major bioactive anticancer component. Specifically, thymidine phosphorylase (TP) converts capecitabine to 5-FU. It is observed that tumor cells produce more TP than normal cells, which underscores the therapeutic preference of capecitabine as an antitumor drug in view of the enhanced localization of 5-FU to tumor cells [14]. Additionally, capecitabine exists in a convenient oral dosage form for administration to experimental animals such as rats.

This study assessed the prophylactic hepatoprotective and anti-HCC effects of a *C. limon* fruit peel extract (LPE) by using experimentally induced HCC in rats. HCC burden was assessed histologically using PAS-stained liver sections. Response to LPE treatment was assessed by monitoring survival rate, body weight changes, and biochemically monitoring *α*-fetoprotein, C-reactive protein, prothrombin time, albumin, and other liver markers (ALT, AST, ALP, GGT, and bilirubin). Interestingly, LPE treatment abated CCl_4_-induced hepatocarcinogenesis.

## 2. Materials and Methods

### 2.1. Chemicals and Reagents

Chemicals and reagents used in the study included carbon tetrachloride (CCl_4_), olive oil, 70% ethanol, chloroform, ammonium hydroxide, hydrochloric acid (Thermo Fisher Scientific, Massachusetts, USA), and stains for histology (periodic acid and Schiff reagent). All the chemicals and reagents used in this study were of analytical grade.

### 2.2. Collection, Identification, and Authentication of *C. limon* Fruits

Two bags (30 kg) of *C. limon* fruits were purchased on the 15^th^ of April 2022 from Jukwa, a small town surrounded by a number of farming villages located about 30 km from the Cape Coast Metropolis. The accurate scientific name of *C. limon* and its synonyms (S1) were verified from http://www.worldfloraonline.org/(https://wfoplantlist.org/plant-list/taxon/wfo-0001133139-2022-12?matched_id=wfo0000608112&page=1). The fruit samples of *C. limon* were identified and authenticated by a curator at the Herbarium, School of Biological Sciences, University of Cape Coast, where a voucher specimen (AB001/2022) was deposited.

### 2.3. Preparation of *C. limon* Fruit Peel Extract (LPE)

The *C. limon* fruits were properly washed under running tap water to wash off all contaminants. The fruits were peeled, and the peels were shade-dried for three weeks. The dried peels were then pulverized using an electric blender into a fine powder (200 g) and stored in an air-tight container. The dry powdered peel sample was extracted with 70% ethanol, and the ethanol was retrieved using the Soxhlet extraction as previously described [16]. The resultant *C. limon* peel extract (LPE) was dried in a desiccator with silica gel. The final extract was weighed, and the weight was used in estimating the % yield of LPE.

### 2.4. Animal Acquisition and Husbandry

Thirty healthy adult male Sprague-Dawley rats (8-10 weeks old) weighing between 150 and 200 g were purchased from the Centre for Plant Medicine Research (CPMR), Mampong-Akwapim, and kept at the animal house of the CPMR. The rats were kept at ambient conditions of temperature, humidity, pressure, and 12 h light/12 h dark cycle. The rats were housed in aluminum cages (320 × 202 × 135 mm) with sawdust bedding, and the beddings were changed regularly. The rats had free access to rodent chow (Grower Mash, Sankofa) and water *ad libitum*. All rats were allowed to acclimatize with the laboratory conditions for two weeks before the commencement of experiments. All animal experiments and procedures used in this study were in full compliance with standard institutional (UCCIRB/CHAS/2022/90), national, and international guidelines (Guide for the Care and Use of Lab Animals, NIH publication No. 85-23) regarding the use of animals in scientific experimentation.

### 2.5. Experimental Design

**Figure 1:**
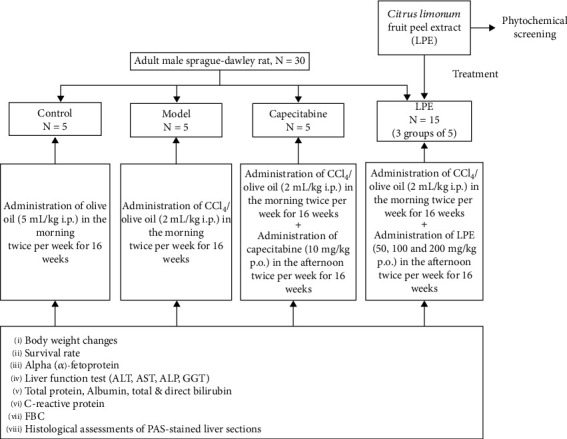
An illustration of the experimental design and dosing schedule for the study. AST: aspartate transaminase; ALT: alanine transaminase; ALP: alkaline phosphatase; GGT: gamma-glutamyltransferase; LPE: *Citrus limon* fruit peel extract; *ip*: intraperitoneal; *po*: per oral.

Thirty (30) adult male Sprague-Dawley rats were randomly assigned into six groups of five rats each. Rats in the various groups were treated for 16 weeks as shown in Figure 1.

### 2.6. Body Weight Measurement

The body weight of rats was measured on a weekly basis, and doses were adjusted to reflect body weight changes. Also, the final body weight of all rats was taken before they were euthanized, and the mean change in body weight was determined for each group.

### 2.7. Blood Collection and Biochemical and Hematological Assessments

Venous blood was collected from cardiac puncture into well-labelled EDTA and gel separator tubes for hematological (full blood count) assessments by using Sysmex XP-300 analyzer and biochemical (liver, kidney, and alpha-fetoprotein test) assessments using Mindray BS-230 auto analyzer. Also, prothrombin time was measured by using a Wondfo Finecare analyzer.

### 2.8. Liver Isolation and Fixation

Livers were surgically removed from randomly selected representative rats (*n* = 3) from each group, and the fresh organ weight was measured. Subsequently, livers were fixed in 10% formalin for histological assessments.

### 2.9. Liver Histology

Paraffin sections (5 mm thick) of buffered formalin-fixed liver samples were stained with the Harris hematoxylin and eosin to study the liver histologic structure of the control, model, and LPE-treated rats. The slides were then mounted using a mixture of distyrene, plasticizer, and xylene (DPX) and covered with a coverslip. The slides were viewed under a light microscope, and images were captured.

### 2.10. Semiquantitation of Glycogen Storage by Recovered Hepatocytes

Photomicrographs of five fields devoid of portal triads or central veins were captured. Each photomicrograph was uploaded into an ImageJ console, and five subfields (upper right, upper left, lower right, lower left, and center panels) were randomly sectioned using the duplicate function. Each subfield was used for the image analysis as follows. In ImageJ, each subfield was split into color components (red, blue, and green) using user-defined vectors. Under the edit function, the red component of the processed image was inverted. The inverted image was subjected to automatic thresholding, and the percentage threshold was recorded.

### 2.11. Data Analysis

GraphPad Prism (version 8.4.3.686 for Windows) and StataSE (version 419.12.1.875) were used to analyze data. Data was presented with descriptive analysis as mean ± standard deviation. Differences between means for the various treatment groups were statistically analyzed using analysis of variance (ANOVA) followed by post hoc analysis. *P* ≤ 0.05 was considered statistically significant in all analyses.

## 3. Results

### 3.1. Phytochemical Compositions of LPE

Standard phytochemical methods were used to screen for specific secondary plant metabolites present in LPE (Table 1).

### 3.2. Effect of LPE Treatment on Body Weight and Survival Rate of Rats

**Figure 2:**
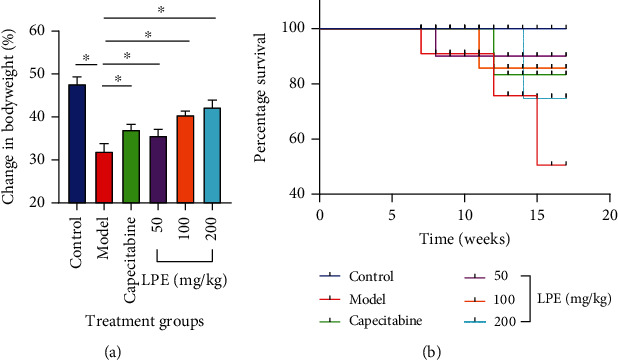
Effect of LPE treatment on body weight and survival rate of rats over 16 weeks of treatment. Mean change in body weight (a) and survival rate (b). Each bar is the mean ± SD, *n* = 3. Control group received olive oil (5 mL/kg *ip*) in the morning twice a week for 16 weeks. The model group received CCl_4_/olive oil (2 mL/kg *ip*) in the morning twice a week for 16 weeks. LPE and capecitabine groups received CCl_4_/olive oil (2 mL/kg *ip*) in the morning and, respectively, LPE (50, 100, and 200 mg/kg *po*) and capecitabine (100 mg/kg *po*) in the afternoon twice a week for 16 weeks. LPE: *Citrus limon* fruit peel extract.

After 16 weeks of treatment, rats in the control group demonstrated significant net gain in body weight over the entire experimental period. Unlike rats in the control group, model rats demonstrated a net loss in body weight. However, treatment of HCC-like rats with LPE improved net body weight gain (Figure 2(a)). At the end of the experimental period control, rats demonstrated 100% survival rate compared to rats in the model group which recorded 20% survival rate. But treatment of HCC-like rats with LPE improved survival rate compared to rats in the model group (Figure 2(b)).

### 3.3. Effect of LPE Treatment on Liver Histology

**Figure 3:**
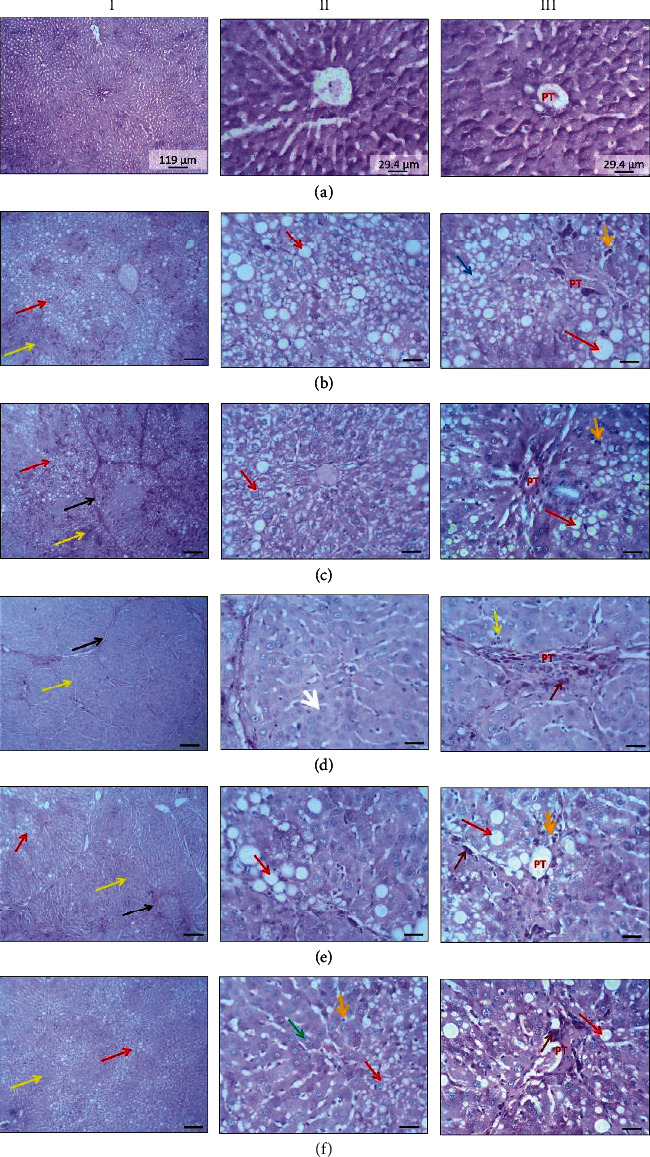
Effect of LPE treatment on periodic acid Schiff- (PAS-) stained liver parenchyma. (a) Control group, (b) model group, (c) capecitabine group, (d) LPE (50 mg/kg *po*), (e) LPE (100 mg/kg *po*), and (f) LPE (200 mg/kg *po*). (I) Liver parenchyma. (II) Centrilobular hepatocytes. (III) Periportal hepatocytes. The control group received olive oil (5 mL/kg *ip*) in the morning twice a week for 16 weeks. The model group received CCl_4_/olive oil (2 mL/kg *ip*) in the morning twice a week for 16 weeks. LPE and capecitabine groups received CCl_4_/olive oil (2 mL/kg *ip*) in the morning and, respectively, LPE (50, 100, and 200 mg/kg *po*) and capecitabine (100 mg/kg *po*) in the afternoon twice a week for 16 weeks. Black arrows represent stromal invasion; red arrows represent hepatocytes exhibiting lipid accumulation; yellow arrows represent liver parenchyma where hepatocytes do not exhibit lipid accumulation. LPE: *Citrus limon* fruit peel extract.

The control group exhibited normal plates of polygonal hepatocytes separated by sinusoids (Figure 3(a)). Compared to the control group, PAS-stained liver sections from model rats demonstrated cytoplasmic fat accumulation in both centrilobular (hepatocytes around central vein) and periportal (hepatocytes around the portal triad) hepatocytes and also hepatocytic focal cytoplasmic basophilia. Also, PAS-stained liver sections from the model rats showed the portal triad area with infiltration of glycogen-positive oval cells and well-differentiated hepatocytes with a hyaline appearance. Additionally, the model group exhibited lymphocytic infiltration (Figure 3(b)). Relative to the model group, the LPE-treated rats exhibited massive stromal invasion and massive lobulation of the liver parenchyma. The lipid accumulations in the periportal and centrilobular hepatocytes were reduced in the LPE-treated rats. Also, LPE-treated rats presented histologically hepatocytes without lipid accumulation. In the LPE (50 mg/kg *po*) group, the hepatocytes exhibited relatively lesser cytoplasmic PAS-staining while LPE-treated rats (100 and 200 mg/kg *po*) exhibited relatively increased cytoplasmic PAS staining (Figures 3(d)–3(f)).

### 3.4. Effect of LPE Treatment on Residual Hepatocyte Glycogen Storage

**Figure 4:**
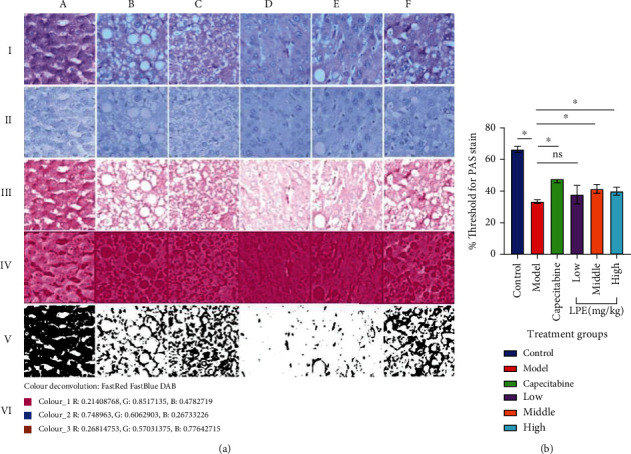
Semiquantitative analysis of glycogen content of hepatocytes. (a) Image processing workflow of micrographs of periodic acid Schiff- (PAS-) stained liver photomicrographs for glycogen quantification. (A) control group, (B) model group, (C) capecitabine group, (D) LPE (50 mg/kg *po*), (E) LPE (100 mg/kg *po*), and (F) LPE (200 mg/kg *po*). (b) Bar graph showing percent threshold for PAS-stained glycogen content in each group. Original micrograph (I). (II, III) Processed images (PAS and hematoxylin, respectively) after color deconvolution using user-defined vectors (VI). (IV) The inverted forms of (II). The control group received olive oil (5 mL/kg *ip*) in the morning twice a week for 16 weeks. The model group received CCl_4_/olive oil (2 mL/kg *ip*) in the morning twice a week for 16 weeks. LPE and capecitabine groups received CCl_4_/olive oil (2 mL/kg *ip*) in the morning and, respectively, LPE (50, 100, and 200 mg/kg *po*) and capecitabine (100 mg/kg *po*) in the afternoon twice a week for 16 weeks. ×400. Each value is the mean ± SD, *n* = 3, ^∗^*P* < 0.05, control vs. model, *n* = 3; ^#^*P* ≤ 0.05, model vs. LPE, *n* = 3. LPE: *Citrus limon* fruit peel extract.

A semiquantitative analysis of the PAS-stained liver sections showed that the glycogen content of the hepatocytes in the livers of the model rats was significantly decreased. Compared to the model group, LPE-treated rats in a dose-dependent manner presented significant improvement in the hepatocyte glycogen content (Figure 4).

### 3.5. Effect of LPE Treatment on Serum *α*-Fetoprotein (AFP) and C-Reactive Protein (CRP) Concentration

**Figure 5:**
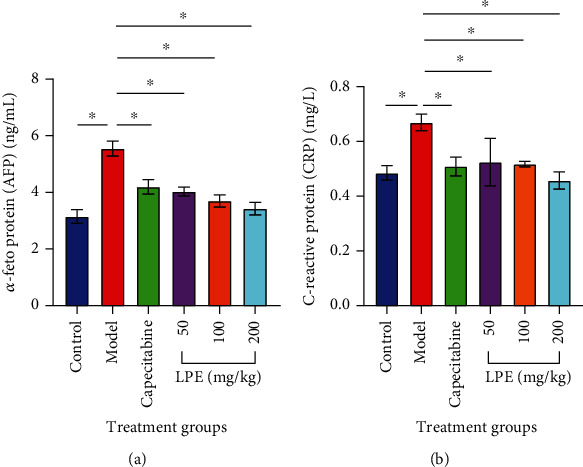
Effect of LPE on serum concentrations of alpha- (*α*-) fetoprotein (AFP) (a) and C-reactive protein (AFP) (b). Control group received olive oil (5 mL/kg *ip*) in the morning twice a week for 16 weeks. The model group received CCl_4_/olive oil (2 mL/kg *ip*) in the morning twice a week for 16 weeks. LPE and capecitabine groups received CCl_4_/olive oil (2 mL/kg *ip*) in the morning and, respectively, LPE (50, 100, and 200 mg/kg *po*) and capecitabine (100 mg/kg *po*) in the afternoon twice a week for 16 weeks. Each value is the mean ± SD, *n* = 3. ^∗^*P* ≤ 0.05, Control vs. model; ^#^*P* ≤ 0.05, LPE and capecitabine vs. model. LPE: *Citrus limon* fruit feel extract.

The serum levels of AFP increased in the model group compared to the control group. Compared to the model group, capecitabine and LPE treatments significantly decreased the serum AFP levels (Figure 5(a)). The serum level of CRP was elevated in the model group compared to the control group. However, compared to the model group, capecitabine and LPE treatments significantly decreased the serum levels of CRP (Figure 5(b)).

### 3.6. Effect of LPE Treatment on Liver Enzymes

**Figure 6:**
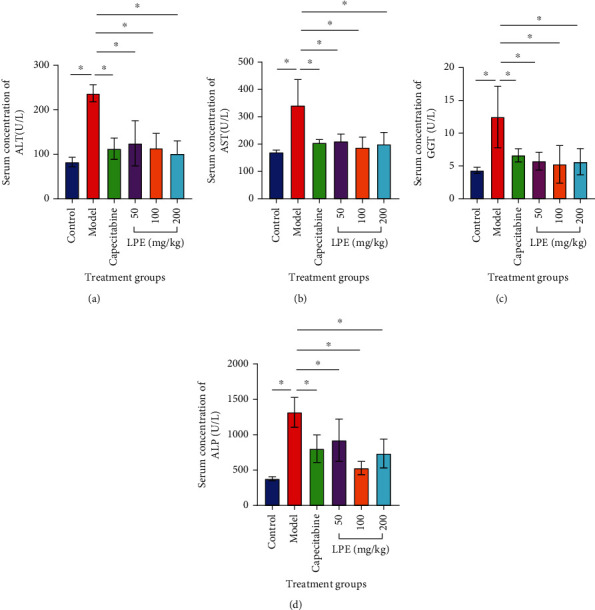
Effect of LPE treatment on serum liver enzymes after 16 weeks of oral exposure of rats to CCl_4_/olive oil. ALT (a), AST (b), GGT (c), and ALP (d). The control group received olive oil (5 mL/kg *ip*) in the morning twice a week for 16 weeks. The model group received CCl_4_/olive oil (2 mL/kg *ip*) in the morning twice a week for 16 weeks. LPE and capecitabine groups received CCl_4_/olive oil (2 mL/kg *ip*) in the morning and, respectively, LPE (50, 100, and 200 mg/kg *po*) and capecitabine (100 mg/kg *po*) in the afternoon twice a week for 16 weeks. Each value is the mean ± SD, *n* = 3. ^∗^*P* ≤ 0.05 (control vs. model); ^#^*P* ≤ 0.05, model vs. LPE. ALT: serum alanine transaminase; ALP: serum alkaline phosphatase; AST: serum aspartate transaminase; GGT: serum glutamyltransferase; LPE: *Citrus limon* fruit peel extract.

The serum concentrations of AST, ALT, ALP, and GGT were elevated in the model group compared to the control group. Regarding ALT, LPE treatment resulted in decreased levels of all of the liver injury parameters measured (Figure 6).

### 3.7. Effect of LPE Treatment on Hematological Parameters

**Figure 7:**
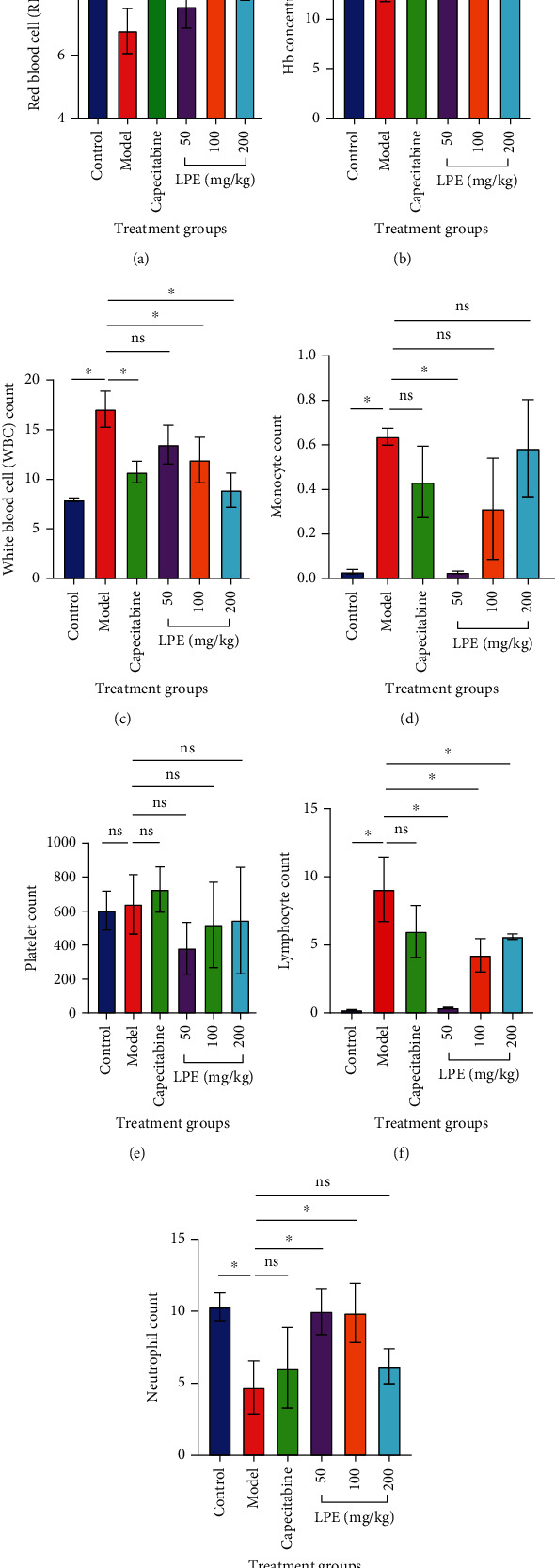
Effect of LPE treatment on full blood count after 16 weeks of oral exposure of rats to CCl_4_/olive oil. RBC (a), hemoglobin (b), WBC (c), monocyte (d), platelets (e), lymphocytes (f), and neutrophil (g). The control group received olive oil (5 mL/kg *ip*) in the morning twice a week for 16 weeks. The model group received CCl_4_/olive oil (2 mL/kg *ip*) in the morning twice a week for 16 weeks. LPE and capecitabine groups received CCl_4_/olive oil (2 mL/kg *ip*) in the morning and, respectively, LPE (50, 100, and 200 mg/kg *po*) and capecitabine (100 mg/kg *po*) in the afternoon twice a week for 16 weeks. Each value is the mean ± SD, *n* = 3. ^∗^*P* ≤ 0.05, control vs. model; ^#^*P* ≤ 0.05, model vs. LPE. RBC: red blood cells; Hb: hemoglobin; WBC: white blood cell; LPE: *Citrus limon* fruit peel extract.

White blood cell (WBC) count was increased in the model group relative to the control group. Also, monocyte and lymphocyte counts were increased in the model group compared to the control group. However, neutrophil count decreased in the model group compared to the control group. Additionally, Hb and RBC counts decreased in the model group compared to the control, whereas platelet count was comparable. Compared to the model group, the WBC count in the LPE group decreased in a dose-dependent manner. Hemoglobin, RBC, neutrophil, and platelet counts improved in the LPE treatment group compared to the model group. Lymphocyte and monocyte counts decreased in the LPE group compared to the model group. With respect to the Capecitabine group, the neutrophil and platelet counts were increased relative to the model group. The Hb, RBC, WBC, lymphocyte, and the monocyte counts were slightly decreased compared to the model group (Figure 7).

### 3.8. Effect of LPE Treatment on Biosynthetic and Metabolic Functions of Liver

**Figure 8:**
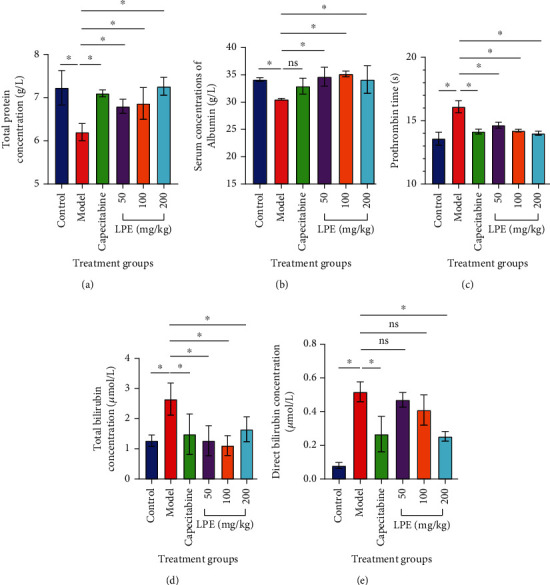
Effect of LPE treatment on biosynthetic function of livers after 16 weeks of oral exposure of rats to CCl_4_/olive oil. (a) Serum total protein, (b) serum albumin, (c) prothrombin time, (d) total bilirubin, and (e) direct bilirubin. The control group received olive oil (5 mL/kg *ip*) in the morning twice a week for 16 weeks. The model group received CCl_4_/olive oil (2 mL/kg *ip*) in the morning twice a week for 16 weeks. LPE and capecitabine groups received CCl_4_/olive oil (2 mL/kg *ip*) in the morning and, respectively, LPE (50, 100, and 200 mg/kg *po*) and capecitabine (100 mg/kg *po*) in the afternoon twice a week for 16 weeks. Each value is the mean ± SD, *n* = 3. ^∗^*P* ≤ 0.05, control vs. model; ^#^*P* ≤ 0.05, model vs. LPE. ns: not significant; LPE: *Citrus limon* fruit peel extract.

Serum total protein and albumin were within the normal concentration range for the control group. Also, prothrombin time was low and within range for the control group. Unlike the control group, model rats presented decreased total protein and albumin concentrations (Figures 8(a) and 8(b)). Prothrombin time increased among the model group compared to control (Figure 8(c)). Compared to the model group, the serum total protein concentration for LPE (50, 100, and 200 mg/kg *po*) treatment groups marginally increased. Compared to the model group, serum albumin concentration for LPE-treated rats increased. Compared to the model group, prothrombin time decreased in LPE-treated rats (Figure 8(c)). Compared to the control group, serum total bilirubin and direct bilirubin were elevated in model rats. Unlike the model group, LPE-treated rats presented decreased serum total and direct bilirubin (Figures 8(d) and 8(e)).

## 4. Discussion

*C. limon* fruit peel extract (LPE) has demonstrated prophylactic hepatoprotective and anti-HCC effects in a rat model of HCC-like phenotype induced by a mixture of CCl_4_ and olive oil. The mechanism involved improvement of all the major structural and functional attributes of the liver, i.e., metabolic, biosynthetic, and detoxification functions. The present finding corroborates earlier reports which indicated that *Citrus* plants of which *C. limon* is a major species harbor diverse secondary plant metabolites with anti-inflammatory, antioxidant, and antiproliferative properties [22, 23]. Inflammation-mediated cancer, HCC, was established in rats using CCl_4_, a common hepatotoxin and a procarcinogen [24]. Mechanistically, upon oral exposure of CCl_4_ to rats, hepatic enzymes specifically CYP2E1 (one of the cytochrome P450 isoenzymes) convert CCl_4_ into trichloromethyl peroxyl radical (CCl_3_OO^−^), the active hepatotoxic intermediate from CCl_4_ metabolism [24, 25]. Trichloromethyl peroxyl radical induces lipid peroxidation and oxidative stress as a result of the generation of endogenous lipid radicals from unsaturated fatty acids within membranes of hepatic cells [24]. When the liver damage due to the generation of endogenous lipid radicals proceeds without treatment, it sets in motion a number of pathological transformations leading to the establishment of HCC-like phenotypes in rats [26]. Thus, to assess the prophylactic hepatoprotective and anti-HCC effects of LPE, three functional hallmarks of the liver, namely, metabolic, biosynthetic, and detoxification were monitored using endpoints such as survival rate, body weight changes, gross liver anatomy, liver histology, biochemical parameters (*α*-fetoprotein, C-reactive protein, albumin, total protein, prothrombin time, AST, ALT, ALP, GGT, and bilirubin), and hematological assessments.

Survival rate is commonly used as a major endpoint measure to assess the efficacy of investigational crude drugs in experimental HCC induced by CCl_4_ and diethylnitrosamine [27–29]. Survival rates determined in experimentally induced HCC in rats after crude drug interventions provide important information for future human studies on such crude drugs. In this study, the model group (rats induced with HCC without treatment) recorded a survival rate of 20% compared to 100% survival rate recorded in the control group. But, LPE (induction of HCC and treatment with LPE at the same time) treatment improved the survival rate to 78%, 85%, and 90% for the respective dose levels of LPE (50, 100, and 200 mg/kg *po*), and these observations were corroborated by results on body weight changes as well as liver histology. LPE-treated rats gained net body weight compared to the model group where there was loss of body weight (Figure 2) indicative of the potential of LPE treatment in mitigating the burden of HCC in rats.

Liver biopsy is used to confirm the diagnosis of HCC and also used to assess the efficacy of interventions [30]. The liver is made up of hexagonally shaped acini, consisting of a portal triad located at the apices, and plates of polygonally shaped hepatocytes separated by capillary sinusoids that drain into a centrally located vein in the acinus [31]. PAS-stained liver sections from model rats demonstrated in both centrilobular and periportal hepatocytes, a large vacuolar fatty accumulation that displaced the nucleus to the periphery (Figure 3), indicative of the structural and functional impairment of hepatocytes [32]. Carbon tetrachloride induces steatosis by a number of mechanisms including increased fatty acid and triglyceride synthesis, synthesis of cholesterol and phospholipids from acetate, and reduced beta-oxidation of fatty acids [33]. Additionally, model rats presented necrotic hepatocytes and nodule formations which were diffused. However, LPE treatment attenuated the CCl_4_-induced steatosis, lipid accumulation in periportal hepatocytes, and centrilobular hepatocytes. This observation suggests that LPE treatment could attenuate CCl_4_-induced hepatocyte necrosis and also improve recovery of necrotized hepatocytes in the portal triad region. Glycogen is specifically stained by PAS; therefore, the glycogen storage potential of the hepatocytes was assessed using a semiquantitation of PAS-stained liver sections from all the groups. The semiquantitation showed that unlike LPE-treated rats, PAS-stained liver sections from model rats demonstrated low glycogen storage, possibly due to increased lipogenesis which was evident from the increased fat accumulation in the periportal and centrilobular hepatocytes (Figure 4). The histological observations which showed that LPE treatment could abate the degree of hepatocyte necrosis and neoplastic transformation were supported by the results from the biochemical assessments such as ALP and CRP.

Alpha- (*α*-) fetoprotein (AFP) is an oncofetal glycoprotein used as one of the standard tumor markers for the diagnosis of HCC as well as a marker for efficacy monitoring of HCC treatment [34]. Although AFP is produced by a number of tissues in the body, the sum of AFP contributions from all these tissues is insignificant compared to that produced by hepatocytes of the fetus during development in the mother's womb. Thus, the fetal liver is the major source of AFP. Therefore, AFP levels normally elevate in maternal blood but decline naturally after birth until the levels reach basal physiological levels. Adult livers lose the ability to produce AFP but damage to hepatocytes during chronic liver injury such as CCl_4_-induced HCC results in conferring the ability to produce AFP on regenerated and necrotized hepatocytes; therefore, AFP elevates in liver disease including HCC [35]. In this study, model rats recorded elevated serum AFP levels confirming the establishment of HCC in rats. But LPE treatment significantly decreased AFP levels comparable even to that of the control and capecitabine groups (Figure 5). This emphasizes that LPE could recover damaged hepatocytes and truncate CCl_4_-mediated DNA damage to hepatocytes as well as abate liver inflammation. HCC is an inflammation-mediated cancer; therefore, a number of acute and chronic phase inflammatory markers help in its diagnosis as well as monitoring of efficacy of interventions [36].

C-reactive protein (CRP) is a pentameric protein synthesized by the liver in response to acute inflammatory insults to the liver; therefore, it is used as an acute phase inflammatory marker [37]. Additionally, liver function test was used to assess liver inflammation. Damage to liver cells leads to leakage of liver enzymes (AST, ALT, ALP, and GGT) into circulation which causes serum enzyme elevations that are used routinely in liver function test [38]. Model rats presented elevated CRP as well as elevated AST/ALT and ALP/GGT that predicted hepatocellular and hepatobiliary damage, respectively (Figure 6). This observation corroborated with the degree of derangements that occurred in the liver parenchyma and hepatobiliary system of model rats. However, LPE treatment reversed this trend of observations in a dose-dependent manner highlighting the probable anti-inflammatory properties of LPE reported earlier [39]. In support, the hematological assessments (Figure 7) show that the prophylactic hepatoprotective and anti-HCC effects of LPE could be mediated through an anti-inflammatory mechanism. Unlike LPE-treated rats, model rats which were subjected to CCl_4_-induced liver inflammation over a period of 16 weeks without treatment presented elevated WBC, monocyte, and lymphocyte counts but decrease in neutrophil count; meanwhile, such hematological profiles are directly associated with neutropenia and thrombocytopenia which are mostly reported in cancer patients [40].

The structural and functional integrity of the liver can be assessed by monitoring the metabolic and biosynthetic potential of the liver. The liver biosynthesizes many macromolecules, including clotting factors, albumin, and prothrombin [31]. Albumin, total protein, and prothrombin time were assayed to assess the biosynthetic function of LPE treatment-related recovery of hepatocytes. Unlike LPE-treated rats, model rats presented decreased albumin and total protein levels but increased prothrombin time suggestive of impaired biosynthetic function of the liver (Figures 8(a)–8(c)). The liver is the main metabolizing organ in the body responsible for the biotransformation of toxic agents into inactive forms for elimination from the body. After the breakdown of senescence RBCs by the reticuloendothelial system, the heme moiety is subsequently metabolized by hepatocytes. First, hepatocytes convert unconjugated bilirubin, which is insoluble into conjugated bilirubin, which is soluble and excretable through the hepatobiliary pathway. Liver impairment occurs when the liver is exposed to CCl_4_ causing a buildup of bilirubin in the blood due to impairment of the hepatobiliary elimination pathway secondary to damaged hepatocytes. Expectedly, model rats presented hyperbilirubinemia indicating CCl_4_-induced hepatocyte damage leading to impairment of the hepatobiliary pathway, thus accumulation of bilirubin in the blood. However, LPE treatment improved the metabolic function of the liver as LPE-treated rats presented decreased direct bilirubin and total bilirubin levels compared to that of model rats (Figures 8(d) and 8(e)). The prophylactic hepatoprotective and anti-HCC effects of LPE reflect its phytochemical composition.

Bioactivity of plant extracts is attributable to their phytochemical composition and diversity [7]. It was observed that anti-inflammatory, antioxidant, and anticancer properties of all the identified secondary plant metabolites in LPE (Table 1) have been demonstrated in other *Citrus* plants. For instance, flavonoids [41], alkaloids [42, 43], terpenoids [44, 45], saponins [46], and tannins [47] were identified in other *Citrus* spp. suggesting that the observed phytochemical signature may be a shared hallmark of all the plant species in the genus *Citrus*. HCC is an inflammation-mediated cancer, and theoretically, anti-inflammatory agents are expected to abate HCC progression. The prophylactic hepatoprotective and anti-HCC effects of LPE were expected given its phytochemical signature.

The present study could have benefited from a number of inputs. For instance, additional assessment of proinflammatory and tumor biomarkers could have further enriched the study significantly given that HCC is an inflammation-related cancer. This study could have benefited from bioassay-guided isolation of specific fractions or components of LPE. Also, this study could have used specific humanized hepatoma cell lines such as HepG2 cells to further elaborate the mechanism of anti-HCC action of LPE as well as other cancer hallmarks including cell proliferation, invasion, and cytotoxicity. Notwithstanding, the present findings provide a solid rationale for further incremental studies on LPE addressing the above limitations.

## 5. Conclusion

*C. limon* fruit peel extract abates CCl_4_-induced hepatocarcinogenesis by attenuating liver inflammation and improving metabolic, biosynthetic, and detoxification functions of the liver. The prophylactic hepatoprotective and anti-hepatocarcinogenic effects of LPE are attributable to its phytochemical composition. This finding provides a background for further incremental studies on potential anticancer bioactive compounds from *C. limon* fruit peels.

## Figures and Tables

**Table 1:** Phytochemical composition of LPE.

Secondary plant metabolites	Phytochemical test	Reference	Results
Alkaloids	Dragendorff's test	[17]	+
Tannins	FeCl_3_	[18]	+
Flavonoids	Sodium hydroxide test	[19]	+
Terpenoids	Salkowski's test	[20]	+
Saponins	Foam test	[21]	+

+ = qualitatively detected; LPE = *C. limon* fruit peel extract.

## Data Availability

Data set is available at https://ir.ucc.edu.gh/xmlui/handle/123456789/8594.

## Abbreviations

AFP: Alpha- (*α*-) fetoprotein
ALT: Alanine transaminase
ALP: Alkaline phosphatase
ANOVA: Analysis of variance
AST: Aspartate transaminase
CCl_4_: Carbon tetrachloride
CPMR: Centre for Plant Medicine Research
CRP: C-reactive protein
LPE: *Citrus limon* fruit peel extract
*C. limon*: *Citrus limon* L.
DPX: Distyrene, plasticizer, and xylene
DRIC: Directorate of Research, Innovation and Consultancy
GGT: Gamma-glutamyltransferase
Hb: Hemoglobin
HCC: Hepatocellular carcinoma
*ip*: Intraperitoneal
*po*: Per oral
PAS: Periodic acid Schiff
RBC: Red blood cell
WBC: White blood cell.

## Supplementary Materials

Supplementary Materials S1 Synonyms of *Citrus limon* (L.) Osbeck. culled from World Flora Online.
