# Supplementary material for: Citrus limon (L.) Osbeck Fruit Peel Extract Attenuates Carbon Tetrachloride-Induced Hepatocarcinogenesis in Sprague-Dawley Rats
Source: Biomed Res Int. 2024 Jan 2;2024:6673550. doi: 10.1155/2024/6673550 (PMC10776197; doi:10.1155/2024/6673550)
Supplement: Supplementary Materials — S1 Synonyms of Citrus limon (L.) Osbeck. culled from World Flora Online. [file 6673550.f1.pdf]

S1. Synonyms of *Citrus limon* (L.) Osbeck

NAME	AUTHOR	PROTOLOGUE	NOMENCLATOR	WFO LINK
Citrus adamii	Risso	Fl. Nice : 85 (1844)		wfo-0000607892
Citrus aurantium subsp. *bergamia*	(Risso & Poit.) Engl.	Nat. Pflanzenfam. 3(4): 198 1896	—	wfo-0000607911
Citrus aurantium var. *mellarosa*	(Risso) Engl.	Nat. Pflanzenfam. 3(4): 198 1896	—	wfo-0000607926
Citrus aurata	Risso	Hist. Nat. Prod. Eur. Mérid. 1: 409 (1826)		wfo-0000607943
Citrus bergamia	(Risso) Risso & Poit.	Hist. Nat. Orangers : t. 53-56 (1819)		wfo-0000607952
Citrus bergamia subsp. *mellarosa*	(Risso) D.Rivera, Obón, S.Ríos, Selma, F.Méndez, Verde & F.Cano	Varied. Tradic. Frutales Río Segura Cat. Etnobot. Cítricos 108. 1998		wfo-0000607953
Citrus bergamota	Raf.	Sylva Tellur. : 141 (1838)		wfo-0000607956
Citrus limetta	Risso	Ann. Mus. Hist. Nat. 20: 195 (1813)		wfo-0000608103
Citrus limetta subsp. *murcica*	S.Ríos, D.Rivera, García Lidón & Obón	Varied. Trad. Frut. Cuenca Río Segura Cat. Etnobot. : 133 (1998)		wfo-0000608104

NAME	AUTHOR	PROTOLOGUE	NOMENCLATOR	WFO LINK
Citrus limettioides	Yu.Tanaka	J. Indian Bot. Soc. 16: 236 (1937)		wfo-0000608105
Citrus limodulcis	D.Rivera, Obón, F.Méndez & S.Ríos	Varied. Trad. Frut. Cuenca Río Segura Cat. Etnobot. : 125 (1998)		wfo-0000608106
Citrus limonelloides	Hayata	Icon. Pl. Formosan. 8: 16 (1919)		wfo-0000608107
Citrus limonia	Osbeck	Reise Ostindien : 250 (1765)		wfo-0000608110
Citrus limonum	Risso	Ann. Mus. Hist. Nat. 20: 201 (1813)		wfo-0000608112
Citrus medica var. *limon*	L.	Sp. Pl. : 782 (1753)		wfo-0000608149
Citrus medica f. *limon*	(L.) M.Hiroe	Forest Pl. Hist. Jap. Islands 1: 218 (1974)		wfo-0000608150
Citrus medica var. *limonum*	(Risso) Brandis	Forest Fl. N.W. India 52 1874	—	wfo-0000608151
Citrus medica subsp. *limonum*	(Risso) Engl.	Nat. Pflanzenfam. 3(4): 200 1896	—	wfo-0000608152
Citrus mellarosa	Risso	Hist. Nat. Prod. Eur. Mérid. 1: 405 (1826)		wfo-0000608163
Citrus meyeri	Yu.Tanaka	Icon. Jap. Citrus Fruits 1: 91 (1946)		wfo-0000608164

NAME	AUTHOR	PROTOLOGUE	NOMENCLATOR	WFO LINK
Citrus volkameriana	Pasq.	Cat. Ort. Bot. Napoli : 29 (1867)		wfo-0000608322
Citrus vulgaris	Ferrarius ex Mill.	–	—	wfo-0000608323
Citrus vulgaris	Ferrarius ex Mill.	Ann. Mus. Natl. Hist. Nat. xx. (1813) 190.		wfo-0000608324
Citrus limonia var. *digitata*	Risso	Hist. Nat. Orang. ? 1813	—	wfo-0000742401
Citrus meyerii	Yu.Tanaka	Icon. Jap. Citrus Fruits 1: 91 (1946)		wfo-0001133156
Citrus medica subsp. *limonia*	(Risso) Hook.f.	Nat. Pflanzenfam. 3(4): 200 1897	—	wfo-0001133259
Citrus aurantium subsp. *bergamia*	(Risso) Wight & Arn.	–	—	wfo-0001259280
Citrus medica f. *limon*	(L.) Hiroë	–	—	wfo-0001284112
Citrus medica var. *limonum*	(Risso) Brandis	–	—	wfo-0001284113
Limon vulgaris	Ferrarius ex Miller	1754	—	wfo-0001284114
Citrus bergamia var. *parva*	Risso	Hist. Nat. Prod. Eur. Mérid. 1: 404 (1826)		wfo-0001380942
Citrus mellarosa var. *vulgaris*	Risso	Hist. Nat. Prod. Eur. Mérid. 1: 405 (1826)		wfo-0001380943

NAME	AUTHOR	PROTOLOGUE	NOMENCLATOR	WFO LINK
Citrus mellarosa var. *plena*	Risso	Hist. Nat. Prod. Eur. Mérid. 1: 406 (1826)		wfo-0001380944
Citrus limon var. *pompia*	Camarda	Quad. Bot. Amb. Appl. 24 (2013): 113 (2015)		wfo-0001381820

Culled from World Flora Online
